# The Algerian Chapter of SARS-CoV-2 Pandemic: An Evolutionary, Genetic, and Epidemiological Prospect

**DOI:** 10.3390/v13081525

**Published:** 2021-08-02

**Authors:** Safia Zeghbib, Balázs A. Somogyi, Brigitta Zana, Gábor Kemenesi, Róbert Herczeg, Fawzi Derrar, Ferenc Jakab

**Affiliations:** 1 National Laboratory of Virology, Szentágothai Research Centre, University of Pécs, 7624 Pécs, Hungary; somogyi.balazs@pte.hu (B.A.S.); brigitta.zana@gmail.com (B.Z.); kemenesi.gabor@gmail.com (G.K.); 2 Institute of Biology, Faculty of Sciences, University of Pécs, 7624 Pécs, Hungary; 3 Genomics and Bioinformatics Core Facility, Bioinformatics Research Group, Szentágothai Research Centre, University of Pécs, 7624 Pécs, Hungary; herczeg.robert@pte.hu; 4 National Influenza Centre, Viral Respiratory Laboratory, Institut Pasteur d’Algérie, Algiers 16000, Algeria; fawziderrar@gmail.com

**Keywords:** SARS-CoV-2, phylogeny, phylogeography, haplotype network analysis, selection pressure, evolution, mutations, transmission route

## Abstract

To explore the SARS-CoV-2 pandemic in Algeria, a dataset comprising ninety-five genomes originating from SARS-CoV-2 sampled from Algeria and other countries worldwide, from 24 December 2019, through 4 March 2021, was thoroughly examined. While performing a multi-component analysis regarding the Algerian outbreak, the toolkit of phylogenetic, phylogeographic, haplotype, and genomic analysis were effectively implemented. We estimated the Time to the Most Recent Common Ancestor (TMRCA) in reference to the Algerian pandemic and highlighted the multiple introductions of the disease and the missing data depicted in the transmission loop. In addition, we emphasized the significant role played by local and international travels in disease dissemination. Most importantly, we unveiled mutational patterns, the effect of unique mutations on corresponding proteins, and the relatedness regarding the Algerian sequences to other sequences worldwide. Our results revealed individual amino-acid replacements such as the deleterious replacement A23T in the *orf3a* gene in Algeria_EPI_ISL_418241. Additionally, a connection between Algeria_EPI_ISL_420037 and sequences originating from the USA was observed through a USA characteristic amino-acid replacement T1004I in the *nsp3* gene, found in the aforementioned Algerian sequence. Similarly, successful tracing could be established, such as Algeria/G37318-8849/2020|EPI_ISL_766863, which was imported from Saudi Arabia during the pilgrimage. Lastly, we assessed the Algerian mitigation measures regarding disease containment using statistical analyses.

## 1. Introduction

Historically, Severe Acute Respiratory Syndrome (SARS) emergence dates back to 2002, and the Middle East Respiratory Syndrome (MERS) epidemic erupted in 2012. In late 2019, the third highly pathogenic human coronavirus was first identified in Wuhan, China, and considered the epicenter cause of a pneumonia outbreak [1]. The novel virus was aptly identified as a severe acute respiratory syndrome coronavirus 2 (SARS-CoV-2) and the primary cause of the coronavirus disease 19 (COVID-19) [2]. Owing to globalized travel, it subsequently spread worldwide and was declared a pandemic by the World Health Organization (WHO), and today is considered a major public health concern [3]. Similarly to MERS coronavirus and SARS coronavirus, the SARS-CoV-2 belongs to the *betacoronavirus* genus and *Sarbecovirus* subgenus, and is related to SARS coronavirus with roughly 80% identity at the nucleotide level [1]. Moreover, despite different hypotheses regarding animal reservoirs and/or intermediate hosts, or even the lab leak theory, the origin of the pandemic remains blurred [4,5]. 

In Algeria, 25 February 2020, marked the first imported case and was registered in the southern portion of the country, when an Italian employee tested positive. This incident was well contained, and no additional cases were reported until the beginning of March 2020, as two more cases were recorded following contact with a family member visiting from France. These cases are considered the onset of the first outbreak in Blida, located in Northern Algeria [6]. In the present study, we aim to understand the dynamics associated with the transmission of all the Algerian SARS-CoV-2 sequences and characterize the identified Algerian SARS-CoV-2 genomes. Notably, this is of significant importance regarding disease containment and both vaccine and drug development [7]. Therefore, a dataset representing ninety-five SARS-CoV-2 sequences comprising a total of twenty-nine Algerian sequences deposited and freely available from the GISAID database was analyzed [8]. To effectively manage the sequencing procedure, we employed the Beast v1.10.4 package for evolutionary and phylogeographic investigations and, additionally, implemented POPART software to create a haplotype network analysis to demonstrate multiple introductions, local transmissions and to further understand the spread and evolution of the disease [9,10]. Moreover, various programs were used for further genome exploration. The results emphasized multiple disease introductions to Algeria and highlighted the role of local and international travels in disease propagation. We noted a mutational heterogeneity at the nucleotide and protein levels across the Algerian genomes, highlighting both unique and common mutations. Furthermore, mutation-based tracing could be established; for instance, a relationship to the USA sequences was confirmed by identifying the USA characteristic amino-acid replacement T1004I (nsp3) in Algeria_EPI_ISL_420037. Likewise, disease introduction from Saudi Arabi during the pilgrimage could be determined via the pangolin lineage classification. However, missing unsampled data were observed during the analysis and might reflect undiagnosed infections during both the first and the second waves of the pandemic.

## 2. Materials and Methods 

### 2.1. Sequence Selection and Maximum Likelihood Phylogeny

To demonstrate the multiple introductions of SARS-CoV-2 to Algeria, ninety-five sequences originating from the current SARS-CoV-2 pandemic, including twenty-nine Algerian genomes, were retrieved from the GISAID database (Table S1) [8,11]. Sequences were aligned by MAFFT using the L-INS-I parameter and manually inspected in MEGA X [12,13]. Subsequently, a maximum likelihood phylogenetic tree was implemented in the IQTREE web server, under the GTR+I substitution model, with ultrafast bootstrapping following the best substitution model selection [14,15]. Considering the Algerian border closure since mid-March 2020, a dataset comprising only the Algerian sequences (18 complete genomes and 11 partial sequences) was analyzed as mentioned above under a GTR+I substitution model.

### 2.2. Temporal Signal Assessment, Time-Calibrated Phylogeny Reconstruction, and Phylogeographic Analysis in Discrete Space

To effectively assess the clock-likeliness regarding the data, the aforementioned maximum likelihood resultant trees were used as input files in TempEst [16]. A regression analysis of root-to-tip genetic distances against sampling times demonstrated strong positive correlation coefficients r = 0.70 and r = 0.75. At the same time, a moderate association was observed R^2^ = 0.49 and R^2^ = 0.56 for the full and the Algerian datasets, respectively, indicating the suitability of both datasets for a phylogenetic molecular clock analysis. Subsequently, the tip-dated phylogenetic trees were generated using the Beast v1.10.4 package and the GTR+I substitution model under a lognormal uncorrelated relaxed clock model [17,18] Considering population size and growth, the parametric coalescent exponential growth model assuming an exponential increase in the population was used as a prior for both the entire dataset and the Algeria dataset. An additional non-parametric Skyline plot model supposing different effective population sizes for each coalescent interval was applied as prior for the Algerian dataset [19]. The MCMC chains were operational for 100 million generations and sampled every 10,000 generations, with 10% discarded as burn-in. Subsequently, the effective sampling sizes (ESS > 200) were examined using TRACER v1.6.0 [20]. In parallel, the date of the most recent common ancestor (MRCA) regarding the pandemic in addition to the evolutionary rate were estimated for both datasets. Furthermore, the Maximum clade credibility trees (MCC) were annotated employing TreeAnnotator v1.10.4 and visualized in FigTree v1.4.4 [9]. Additionally, both a discrete and a continuous phylogeographic analysis were implemented using Beast v1.10.4. [21]. The samples’ spatial data/location of isolation was used to infer the geographical spreading patterns of the virus in Algeria by combining the Bayesian stochastic search variable selection/BSSVS with a standard symmetric substitution for the discrete diffusion. On the other hand, The Brownian diffusion model assuming a homogeneous diffusion rate over the phylogeny was employed when considering a continuous space. Thereafter, SpreaD3 v0.9.7 software was used to visualize the transmission routes and calculate the Bayes Factor (BF). For this, the MCC trees and the discrete analysis’s log file were respectively used [22].

### 2.3. Genome Investigations

The selective pressure at the protein level was evaluated for each sequence pair, within each of the following genes: *ORF1a*, *ORF1b*, *S*, *E*, *M*, *N*, *ORF3a*, and *ORF8*, through the estimation of the ω ratio representing the rate of the non-synonymous mutation (Ka/dn) to the synonymous mutations (Ks/ds), according to Nei and Gojobori using SNAP v2.1.1 [23,24,25]. When several non-synonymous mutations that promote changes with physiochemically different amino acids occur, they show a tendency to be deleterious to the protein. Thus, they are improbable to become fixed in the population leading to an adverse selection resulting in Ka < Ks (ω < 1). Contrariwise, when advantageous non-synonymous substitutions strike, they are likely to become fixed in the population, and thus amino acid changes in the protein are enhanced (ω > 1). Lastly, we subjugated the Algerian sequences to the genome detective coronavirus typing tool and the CoVsurver mutations App implemented in the GISAID database to highlight variations in the mutational pattern both on the amino-acid and nucleotide levels among them [11,26]. Subsequently, the Cov-glue webserver was used to assess the effect of the amino-acid replacement on the corresponding protein according to Hanada and colleagues amino acid classification and in reliance on both Grantham and Miyata scores [27,28,29,30]. Thereafter, the PredictSNP webserver combining several prediction tools (https://loschmidt.chemi.muni.cz/predictsnp/, accessed on 1 August 2021) served to evaluate the effects of mutations on protein function and disease relation [25,31,32,33,34].

### 2.4. Haplotype Network Analysis

A dataset comprising eighty-four sequences, including the Algerian genomes (the partial Algerian genomes were not included), was subjected to recombination detection analysis using RDP 4 software. Then, the DnaSP v6.12.03 package was applied to estimate insertions or deletions (InDels), recombination and haplotype generation [35]. Subsequently, the median-joining network method implemented in POPART software was employed for the haplotype network analysis with default setting/epsilon = 0 [10,36,37].

### 2.5. Epidemiological Analysis and Preventive Measures Assessment

In summary, to draft an overview encapsulating the evolution of the pandemic in Algeria, the cumulative number of the infected recovered and death cases were collected from the Johns Hopkins University Center for Systems Science and Engineering (2 May 2021) [38]. Subsequently, the linear, exponential, and logarithmic trend lines were compared, and the best model was chosen based on the R^2^ values. Furthermore, the cumulative confirmed cases for each of the forty-eight Algerian cities were collected from the official Algerian Ministry of Health website [39]. Additionally, the population density data regarding all Algerian cities was retrieved from the Wikipedia website [40]. Thereafter, the correlation coefficient was calculated between the density and the number of confirmed cases.

## 3. Results

### 3.1. Evolutionary Phylogenetic and Phylogeographic Analyses

The estimated MRCA regarding the Algerian pandemic was 28 January 2020 [29 October 2019, 29 February 2020] under the skyline coalescent prior, whereas it was estimated to 15 June 2012 [24 November 1999, 27 February 2020] under the exponential coalescent model. The exponential prior was less suitable for the Algerian dataset, and thus only results under the skyline model were considered for downstream analysis. The SARS-CoV-2 evolutionary rate for the global pandemic was 5.4043 × 10^−4^ substitution/site/year [5.2458 × 10^−4^, 8.2507 × 10^−4^].

**Figure 1:**
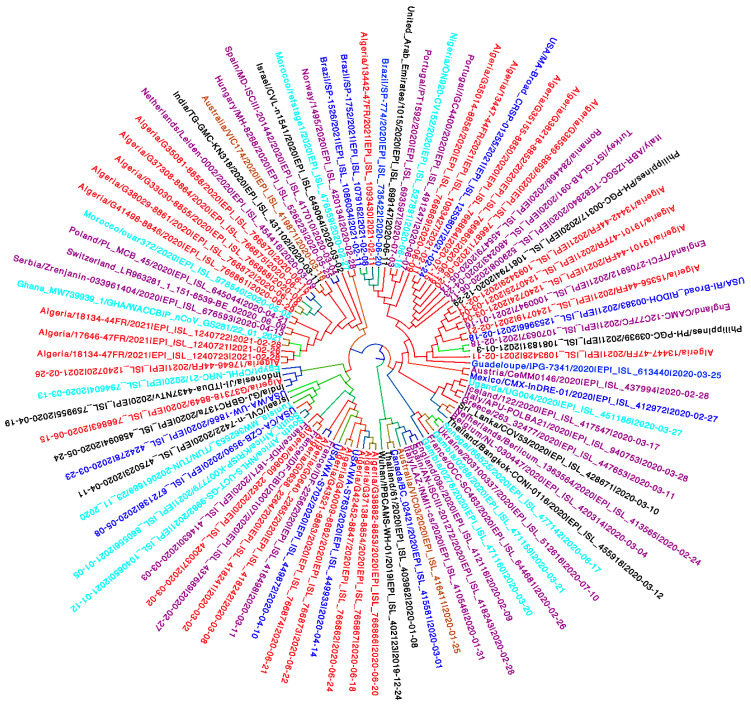
Bayesian phylogenetic trees implemented using BEAST v1.10.4 based on ninety-five genomes sampled worldwide. Colors indicates sampling locations. Blue represents sequences from America; Purple indicates sequences from Europe; Black shows sequences from Asia; Cyan for sequences from Africa; sequences from Australia are labelled in orange and sequences from Algeria are indicated in red.

Furthermore, based on the maximum clade credibility tree (MCC) (Figure 1), Multiple introductions were clearly observed via the interspersion of the Algerian sequences within the phylogenetic tree. This indicates different origins of the SARS-CoV-2 pandemic in Algeria. The enlarged time-dated tree with posterior probabilities is supplemented as Figure S1.

**Figure 2:**
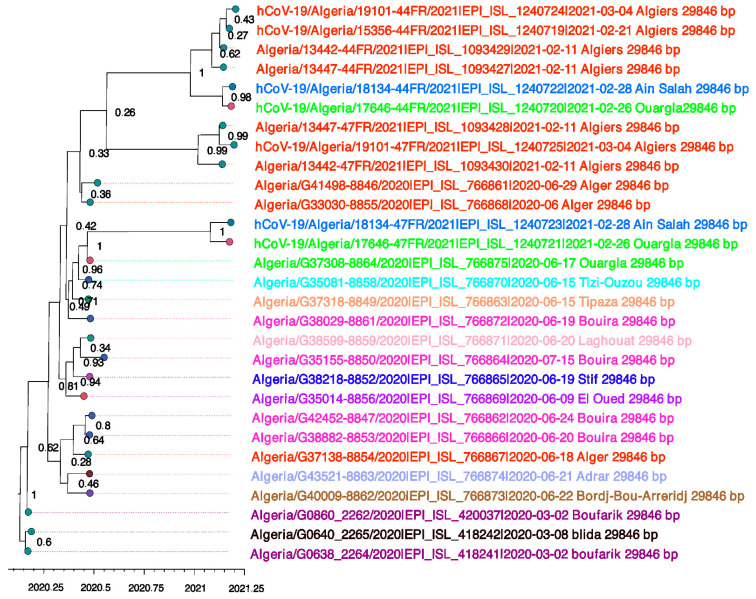
Maximum clade credibility tree. A Bayesian phylogenetic tree reconstructed using twenty-nine Algerian genomes. Posterior probabilities are indicated at each tree node. Branches are time-scaled in decimal years. Sequences are colored according to their sampling location.

Moreover, since Algeria was under a complete lockdown starting from mid-March 2020, all transmissions occurring following this date are either local or a result of Algerian repatriation. However, the impact of domestic travels on the pandemic spread can be perceived (Figure 2). For instance, hCoV-19/Algeria/18134-44FR/2021|EPI_ISL_1240722|2021-02-28 (Ain Salah) formed sister taxa together with hCoV-19/Algeria/17646-44FR/2021|EPI_ISL_1240720|2021-02-26 (Ouargla) with high posterior probability (PP = 98%). Interestingly, these sequences didn’t cluster with sequences from the same provenance, but instead, they formed a monophyletic clade with sequences from Algiers (PP = 1).

**Figure 3:**
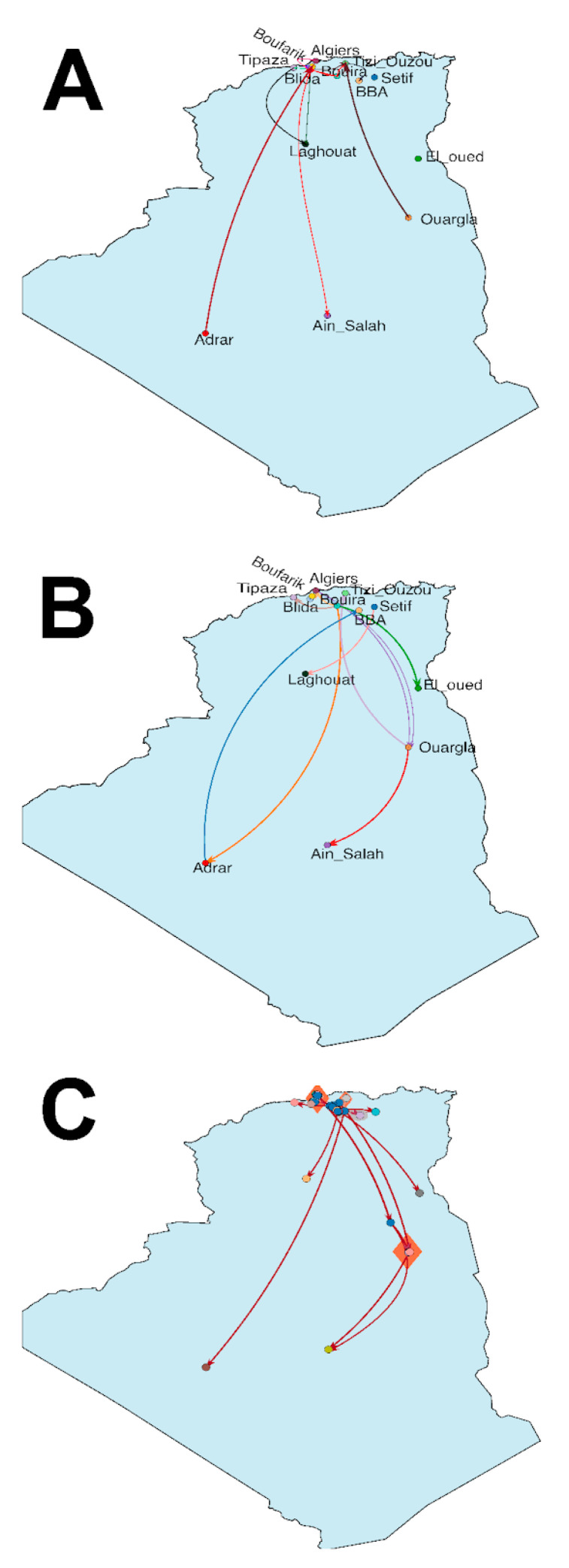
(**A**) Bayes factors for significant discrete transitions. (**B**) SARS-CoV-2 diffusion in discrete space in Algeria. The arrows indicate the spread direction, and they are coloured differently to distinguish different transmission routes. (**C**) SARS-CoV-2 spread in continuous space in Algeria. The arrows indicate the diffusion direction; they are coloured according to posterior probabilities (gradient from blue = 0.3 to red = 0.8–1), polygons display the uncertainty and are coloured according to the posterior probabilities (gradient from blue = 0.3, to orange = 0.8–1), Blue circles indicates internal nodes, (**B**,**C**) different coloured circles indicate the sampling locations.

Likewise, to explore the spread of the SARS-CoV-2 in Algeria, both discrete and continuous approaches were used. Considering the discrete phylogeographic analysis, while relying on the Bayesian stochastic search variable selection /BSSVS and Bayes factor calculation, 14.1 non-zero-rates between cities were identified [95% HPD = 14–16], of which twelve were well-supported migration rates (BF > 3) (Figure 3A). The transmission route started from Boufarik towards Blida and Bouira, then from Bouira to Adrar, Algiers, Setif, Ouargla, El Oued and Tipaza, after that, from Ourgla to Tizi Ouzou. Setif to Laghouat, subsequently from Adrar to Bordj Bou Arreridj, and finally, from Bouira to Ouargla and Ouargla to Ain Salah (Figure 3B). In parallel, a continuous dispersion model was employed to get a more detailed overview regarding the diffusion process based on the reconstruction of the ancestral viral location coordinates assumed from the latitude and longitude of the sampling locations. The estimated diffusion rate was 1620.3 km/year. The root coordinates corresponded to Bouinan in Blida. In parallel, the transmission route started from Blida to Boufarik and back to Blida, following this, Bouira to El-Oued, Tizi Ouzou, and Tipaza, then from Bouira to Sétif, Laghouat, and Adrar, thereafter Bouira and Algiers towards Ouargla, and from Ouargla to Ain-Salah (Figure 3C).

### 3.2. Genome Analyses

**Figure 4:**
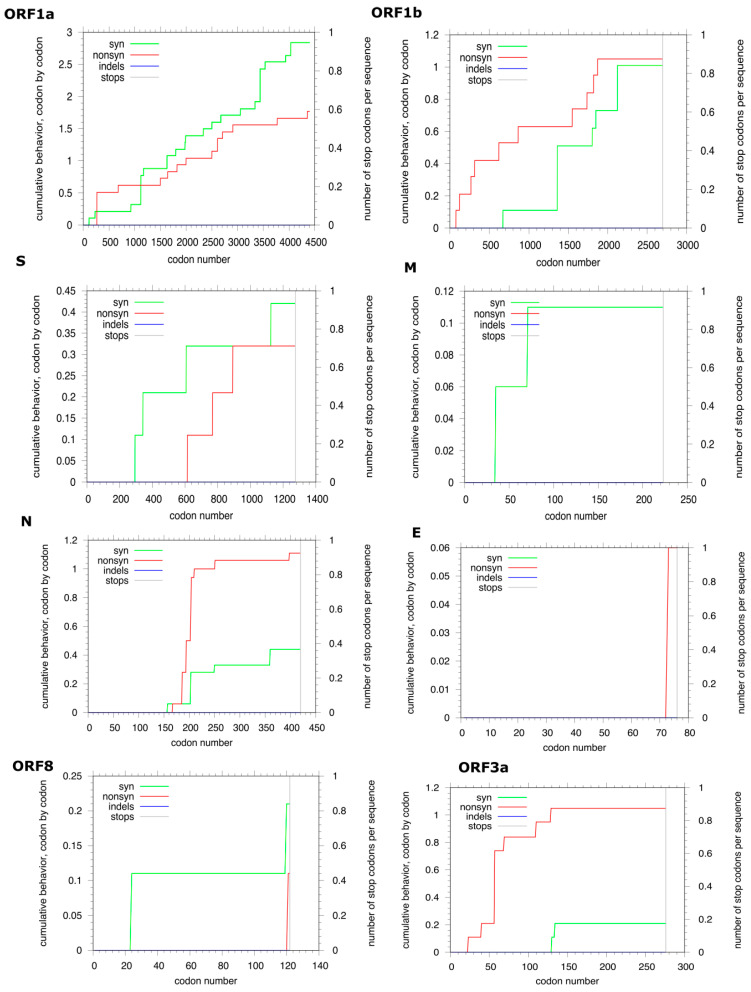
Cumulative behavior of synonymous and non-synonymous substitutions along each of the eight analyzed genes. Accumulation of amino acid substitutions for nineteen sequences comprising the SARS-CoV-2 reference sequence and eighteen complete genomes originating from Algeria are plotted. The x-axis represents codons for the entire coding region of each gene. The y-axis refers to the number of substitutions. The plot for synonymous substitutions is in red, while green depicts the non-synonymous substitutions. The grey vertical lines indicate stop codons.

When relying on both the codon-by-codon cumulative behavior plots for synonymous and non-synonymous substitutions displayed in Figure 4 and the calculated values of the non-synonymous (dn) to synonymous (ds) mutations ratio (ω), the evolutive selection pressure was assessed for each gene. Only the Wuhan reference sequence and the eighteen Algerian complete genomes were considered for this analysis. In the *ORF1a* gene, for the first two-hundred codons, only a moderate rate of synonymous mutations was observed, then it was stationary. At the same time, the non-synonymous rates increased till the codon number one thousand one hundred. Subsequently, both synonymous and non-synonymous rates increased with higher synonymous mutation rates until the end of the coding region. The average of the pairwise (ω) ratio was 0.2. In parallel, in consideration of the *ORF1b* gene, at the beginning of the coding region, only non-synonymous mutations raised till the codon six hundred eighty, from where both synonymous and non-synonymous mutation rates showed an alternance between increase and stationary phases till the stop codon, yet the non-synonymous rates were superior. The dn/ds ratio was equal to 0.22. Across the *S* gene, a raise in the synonymous rates was apparent from codon two hundred eighty with the alternance of sharp increase and stationary phases till codon six hundred, where the non-synonymous rate leveled up with the same alternance pattern and a higher ds rate till codon ninety-two, at this point, a neutral stationary phase was observed (ds = dn) till codon one thousand one hundred sixty where a higher synonymous rate is detected. The average ω ratio was 0.3. Similarly, in reference to the *M* gene, the codon-by-codon cumulative behavior demonstrated a sharp increase of the synonymous rate in the codon position thirty-five, followed by a stagnant rate till codon sixty-nine, after this, a sharp rate increase in codon position seventy, followed by a stationary phase until the end of the coding sequence. Interestingly, non-synonymous mutation cumulations were not observed, and thus the calculated dn/ds ratio was <1 (0.3). As for the *N* gene, a higher non-synonymous rate was observed compared to the synonymous mutation rate. The ω ratio was 0.38. Likewise, the *E* gene displayed only a high non-synonymous mutation rate in the codon seventy-two with a dn/ds ratio equal to 0.08. Regarding the *ORF8* gene, codon positions twenty-four and one hundred sixteen cumulated synonymous mutation rates with a constant rate between the two positions, whereas the codon position one hundred twenty cumulated non-synonymous mutations, the dn/ds was 0.25. Finally, the *ORF3a* displayed a higher cumulation rate of non-synonymous than synonymous mutations with an ω value of 0.33. Overall, all dn/ds values were less than one for all genes indicating negative selection pressure.

Hereafter, based on Rambaut et al. genome lineage attribution and the GISAID clade classification, the Algerian sequences fall within different lineages and clades [8,41]. Additionally, several SNPs were detected in the Algerian sequences compared to the reference sequence (NCBI RefSeq NC_045512). All aforementioned results are summarized in Table 1.

Similarly, amino acid replacements were observed across different genes when compared to the reference sequence. Strikingly, several amino acid replacements with or without an impact on the matching protein were proper to the Algerian genomes (Table 2). Subsequently, the effect of the unique amino acid replacements on the Algerian sequences was first assessed in CoV-GLUE (Data in Table S2). Thereafter, accuracy scores for the PredictSNP webserver are indicated in Table 3 and color-coded according to the mutation type, in which Green indicates a neutral mutation and Red shows a deleterious mutation.

### 3.3. Haplotype Network Analysis

**Figure 5:**
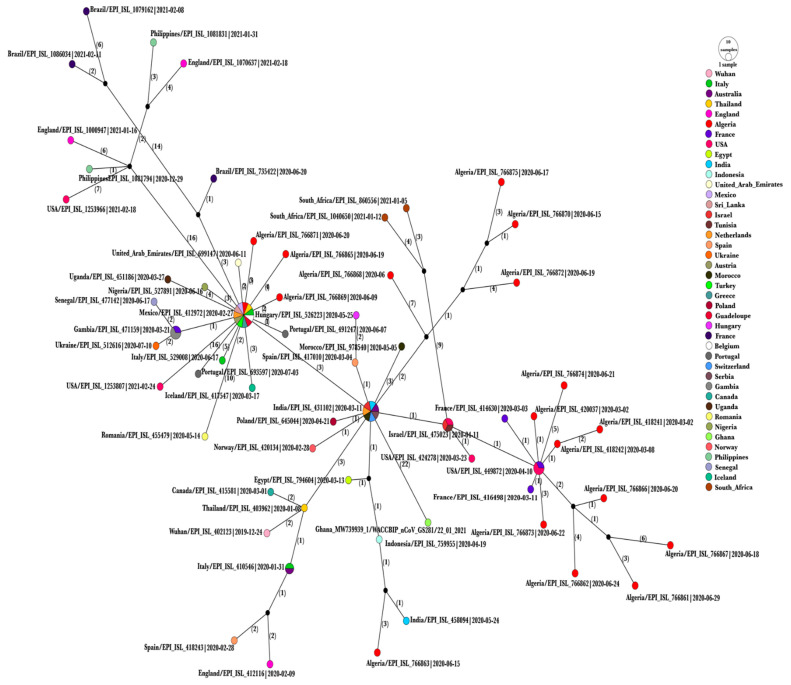
A haplotype network analysis representing eighty-one SARS-CoV-2 genomes. The median-joining algorithm with epsilon = 1 parameter was used in the network construction. ellipses and diameters are proportional to the number of sequences. Mutation steps between haplotypes are represented by the bracketed numbers. All mutations were enclosed. Colors symbolize the different geographic sampling locations.

Based on RDP4 analysis, no recombination events were detected within the dataset. Sixty-one haplotypes were observed. The Algerian sequences are scattered across the network as demonstrated in Figure 5. For instance, Algeria/G35155-8850/2020|EPI_ISL_766864 clustered with sequences from Mexico, Sri Lanka, Austria, Netherlands, Turkey, Italy, Greece, Guadeloupe, Thailand, Belgium and formed one haplogroup. In contrast, each of the seventeen remaining sequences was considered as an individual haplotype. Algeria/G38599-8859/2020|EPI_ISL_766871, Algeria/G38218-8852/2020|EPI_ISL_766865, and Algeria/G35014-8856/2020|EPI_ISL_766869 were directly linked to the previously mentioned haplogroup. Similarly, Algeria/G0638_2264/2020|EPI_ISL_418241 and Algeria/G0640_2265/2020|EPI_ISL_418242 were directly interconnected. On the other hand, Algeria/G37138-8854/2020|EPI_ISL_766867 and Algeria/G41498-8846/2020|EPI_ISL_766861 were joined with the presence of a median vector indication missing unsampled data.

### 3.4. Epidemiological Analysis

**Figure 6:**
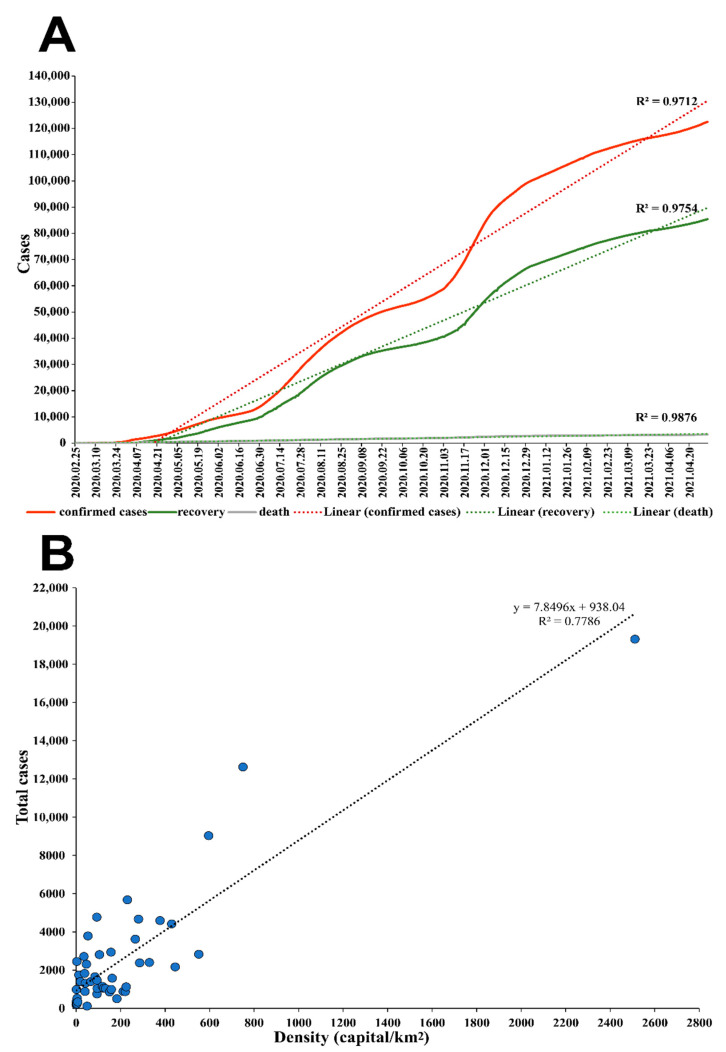
(**A**) Time series plot representing the COVID-19 confirmed cases (Red), recovery (Green), and death (Grey) depicting corresponding R^2^ values. (**B**) Correlation plot between the population density and the total confirmed cases.

The R^2^ values regarding the time series plot of the cumulative confirmed cases were 0.62, 0.97, and 0.97 for the exponential, linear, and logarithmic trend lines, respectively. Likewise, the same results were observed regarding recovery cases. At the same time, the cumulative death cases exhibited the following R^2^ values: 0.77 for the exponential trend line, 0.99 for the linear, and the logarithmic model (Figure 6A). In parallel, the correlation coefficient calculated between the confirmed cases and the population density for each of the forty-eight Algerian cities was 0.78 (Figure 6B).

## 4. Discussion

To investigate the spread of SARS-CoV-2 in Algeria, we performed a thorough analysis of all the complete and partial SARS-CoV-2 sequences available from Algeria (twenty-nine) in addition to sixty-six sequences sampled worldwide.

Our estimations regarding the MRCA of the SARS-CoV-2 Algerian pandemic under a relaxed molecular clock with the skyline model was 28 January 2020 [29 October 2019, 29 February 2020]. These results are coherent, as the restriction measures in Algeria began in mid-March 2020 [6]. The evolutionary rate of the SARS-CoV-2 pandemic in the present study was equal to 5.4043 × 10^−4^ substitution/site/year as of March 2021. In parallel, the substitution rate previously reported early in the pandemic was 1.66 × 10^−3^ in February 2020, whereas 8.99 × 10^−4^ in early August 2020, which is in line with the time-dependent pattern of substitution rates observed in viruses [2,42,43,44].

Moreover, the phylogenetic analysis revealed both multiple disease introductions into Algeria and disease transmissions between cities. Thus, highlighting the impact of international and domestic travels in disease spread. The first three sequenced samples from March 2020 were introduced from France to Algeria as previously demonstrated through contact tracing and phylogenetic analysis, yet; they didn’t cluster together within the current study indicating indirect contamination [6,45]. Likewise, the discrete phylogeographic analysis of the virus expansion in Algeria emphasized between city transmissions, both vertical (from the north to the south and vice versa) and horizontal (within only northern cities, or just southern cities) transmissions were observed. For instance, within the northern part of Algeria from Bouira to Blida (BF 37.19) and Bouira to Tizi Ouzou (BF = 6.01), from the north to the south of the country, Blida to Ain Salah (BF = 5.69,) and from Adrar, a southern city to Boufarik a municipality in the town of Blida in the North of Algeria (BF = 91.69). The continuous phylogeographic analysis gave more details about the route of expansion by reconstructing the ancestral locations of the virus indicated as internal nods. Unsurprisingly, internal nods were placed in municipalities representing crossing points between several cities, such as Djebahia in the city of Bouira, where travelers take breaks. Another example is Hassi Messaoud in Ouargla, where a large oil station employing several international workers is located. The first formally considered coronavirus case in Algeria was detected in an Italian worker from this oil station [6,46]. The phylogeographic results are in accordance with the phylogenetic analysis, emphasizing the importance of local travels and social contact in the spread of the disease. Globally, the ω ratios across all the analyzed coding genes (*ORF1a*, *ORF1b*, *S*, *M*, *N*, *E*, *ORF3a*) for the Algerian sequences in comparison to the reference genomes were inferior to one indicating negative selection. The same results were observed in a similar study on SARS-CoV-2 in a Canadian population conducted by Zhang et al. [47]. In addition, a recent analysis performed on 260,673 whole-genome sequences to study the selection pressure among the coding genes highlighted the rarity of positive selection in SARS-CoV-2 protein-coding genes [48].

Complementary to this, several common non-synonymous mutations were detected among the Algerian sequences. This included T85I in the *nsp2* gene, P423L in the *nsp12* gene, D614G in the *S* gene, and Q57H in the *ORF3a* gene. Notably, these mutations were detected within eighty-four countries and thus considered as positively selected. Moreover, amino-acid replacements in the Spike protein characteristic of the newly identified SARS-Cov-2 variants were also identified. Namely, the H69del, V70del, E484K, Y144del, and Q52R. This was after the repatriation of Algerian nationals from abroad. Following this, Algeria enforced a full lockdown for the second time [46]. Interestingly, characteristic non-synonymous mutations were identified. To cite an example, the T1004I replacement in the *nsp3* gene was detected in the sequence Algeria_EPI_ISL_420037. This mutation was spotted as a unique mutation in the USA in the early stages of the pandemic, in sequences from 19 January 2020 to 15 April 2020 and was not reported elsewhere. We conclude that the individual who contaminated Algeria_EPI_ISL_420037 had either a travel history to the USA or was in contact with an individual who introduced the disease to France originating from the USA [49]. Strikingly, unique non-synonymous amino-acid changes were found. In the sequence, Algeria/EPI_ISL_766874, the amino acid substitution A130V in the *RdRp* gene result in a harmful functional effect on the protein responsible for viral replication. This mutation was first reported in the United Arab Emirates on 12 June 2020. It occurred only in seventy-five samples worldwide in thirteen countries. In Algeria, it was detected on 21 June 2020, since the sample collection dates in all the other locations where this mutation was described ranged from 20 of August 2020 till 19 April 2021, excluding them from originating countries of the disease and thus linking the sample Algeria/EPI_ISL_766874 directly to the sample EPI_ISL_698151 from Abu Dhabi. Likewise, a deleterious mutation results from the non-synonymous amino acid replacement N874H in Algeria/EPI_ISL_766875 in the *NSP12* gene. This amino-acid replacement occurred the first time in Algeria and only in seven samples worldwide. Based on the collection dates, the sequence can be linked directly to the EPI_ISL_557768 genome from England sampled right after the original sequence on 6 July 2020. Similarly, In the accessory gene *ORF3a*, which plays an important role in virulence, infectivity, and virus release, the deleterious mutation A23T was first reported from the USA and sampled sixty-two times in fifteen countries; thus, based on collection dates, the sequence Algeria/ EPI_ISL_766862 is related to sequences from Texas (USA) [50].The last deleterious amino acid replacement, L129F, was found in the *NS3* gene and detected for the first time in Algeria. It occurred in nine hundred ninety-seven samples worldwide. This mutation occurred in the third functional domain of the ORF3a protein (K+ ion channel) and may seriously impact the protein function and consequently the virus phenotype [50]. The circulation of different deleterious mutations is in line with previous reports regarding deleterious mutations in RNA viruses with zoonotic potential. The occurrence of different deleterious mutations simultaneously with the presence of stabilizing mutations may increase virus fitness. This is the case for influenza A/H5N1, which required a combination of mutations to gain airborne transmissibility, of which two were deleterious [51]. However, the strength of the purifying selection is not sufficient to directly eliminate the deleterious mutations after their occurrence; hence they might circulate for a sufficient period to impact the viral infection path [52]. Moreover, deleterious mutations might be used to develop treatment strategies. For instance, provoking a mutational meltdown phenomenon (population extinction) by giving a drug such as Favipiravir, which increases the accumulation rate of harmful mutations, subsequently inducing population collapse [53]. In parallel, three neutral amino acid replacements were identified among the Algerian sequences; this implies no changes in the protein function [54]. The E681D amino acid replacement in the protease gene (*NSP3*) was first acknowledged in the Algerian genome EPI_ISL_766862 and occurring only in three samples worldwide demonstrated disease exportation from Algeria to Austria (EPI_ISL_853900) via the sample collection dates. Furthermore, in Algeria/EPI_ISL_418241, two neutral amino acid replacements were identified. The first in the exonuclease gene (*NSP14*), H26Y amino acid substitution originally discovered for the first time in the aforementioned Algerian sequence and right after in the Greek sequence EPI_ISL_437907, subsequently supporting relatedness of the two genomes. In the envelope gene, the Leucine substitution with Phenylalanine in position seventy-three was first revealed from the Algerian sequence, then reported in 2795 samples worldwide. This mutation was proven earlier to alter the DLLV motif (change to DFLV). Distinctly, it may delay Tight Junction formation and therefore may hypothetically affect viral replication and/or infectivity [55]. The above-mentioned viral mutation fingerprints might help characterize and identify both transmission patterns and superspreaders, as previously demonstrated [56].

Meanwhile, the Algerian genomes fell within five lineages, the lineage A being considered as the root of the pandemic in which many sequences originated from China. All Algerian sequences within this lineage were partial genomes (*S*, *NSP16*) and were characterized with either the B.1.1.7 (UK), B.1.351 (South Africa), or B.1.525 (Nigeria) related mutations. The length of the sequence is one of the biggest drawbacks of an accurate analysis [55]. Furthermore, lineage B.1, a large European clade corresponding approximately to the Italian outbreak, and the clade B.1.1 corresponding to a European lineage with three clear SNPs: G28881A, G28882A, G28883C were also identified amid the Algerian sequences. Similarly, the clade B.1.597 corresponding to sequences Mainly from France was determined. Interestingly, one of the Algeria sequences appertained to the B.1.36 lineage declared for the first time in February 2020 in Saudi Arabia and clustered with both an Indian (PP = 94%) and a Malaysian sequence (PP = 92%). This sequence was isolated from an eighty-two-year-old woman. Undoubtedly, the virus was imported from Saudi Arabia while performing the pilgrimage as no repatriation flights were scheduled in the destination of Malesia and India, unlike Saudi Arabia. These results are in line with reports regarding Algerian repatriation from abroad [46].

Furthermore, as per the results mentioned above, the haplotype network analysis displayed seven median vectors amid the Algerian sequences indicating missing or unsampled data. The multiple introduction theory was clearly visible in the network, confirmed by the heterogeneity of the Algerian haplotypes.

In the present study, we demonstrated the evolution representing the Algerian pandemic in a consistent manner, while simultaneously reflecting the effectiveness of various implemented measures. Moreover, the strong correlation between the number of SARS-CoV-2 confirmed cases and the population density in each Algerian city implies the spread of the virus is primarily dependent on social contact, the awareness of the community, and the respectful compliance regarding social distancing, seeing as how lower infection cases in relatively high population density cities were observed and vice-versa. To cite an illustration, Ouargla, a city located in the southern portion of Algeria, has a population density of 2.63 habitant/km^2^, although the number of confirmed cases is two thousand four hundred fifty-three. Whereases, in Bordj Bou Arreridj, situated in the Northern area of Algeria, the number of confirmed cases is five hundred six cases for 182.76 habitant/km^2^. Overall, the Algerian government’s restriction measures were effective regarding disease containment and prevented catastrophic scenarios such as the Italian one [57]. This is complementary to an epidemiological study conducted to assess the mitigation measures implemented in Algeria in the early SARS-CoV-2 pandemic (dated 26 April 2020), which demonstrated the efficiency based on the basic reproduction number R0 before and after the implementation of the preventive strategy [6].

Overall, we explored the evolutionary, genetic, and epidemiological aspects regarding the Algerian SARS-CoV-2 pandemic, aptly demonstrating the multiple introductions of the disease and the heterogeneity of the genomes. Additionally, our research findings revealed unique amino-acid substitutions by characterizing the mutational patterns and the effect on the corresponding proteins. In addition, some concise tracing could be performed based on both unique mutations and travel history. Statistically, we assessed the effectiveness regarding the mitigation majors implemented against the SARS-CoV-2 pandemic. Admittedly, the main drawback regarding our study was the length of some sequenced genomes and the size of the Algerian data panel. Thus, we emphasized the importance of massive sampling and sequencing in disease comprehension and increased efforts regarding diagnostics, therapy, drug, and vaccine development. Given that Algeria was under complete travel restrictions since 15 March 2020, the number of cases kept increasing, indicating local transmissions. Thus, these local viral variants may potentially represent a distinct strain as previously occurred [9].

## Figures and Tables

**Table 1:** Lineages and clades assigned to the Algerian sequences, in addition to all observed SNPs.

Query	Gisaid Clade	Pangolin Lineage	SNPs	Query	Gisaid Clade	Pangolin Lineage	SNPs
**Algeria/EPI_ISL_766874**	GH	B.1	C601T, C1059T, C3037T, C6255T, C8290T, C10582T, A13693G, C13829T, C14408T, A23403G, C25511T, G25563T, C29025T	**Algeria/EPI_ISL_766861**	G	B.1.597	C3037T, C3619T, C5144T, C10582T, C12367T, C14408T, C17550T, A23403G, C27804T, C28830A
**Algeria/EPI_ISL_766862**	GH	B.1.597	C1059T, C3037T, A4762C, C7765T, C10582T, C14408T, G15327T, A23403G, G25459A, G25563T, C27804T, C28830A	**Algeria/** **EPI_ISL_420037**	GH	B.1	C1059T, C3037T, C5730T, C10582T, C14408T, A23403G, G25563T
**Algeria/EPI_ISL_766866**	GH	B.1.597	C1059T, C3037T, T6199C, C10582T, C14408T, A23403G, G25563T, C25782T, C27804T, C28830A	**Algeria/EPI_ISL_418242**	GH	B.1	C1059T, C3037T, C10582T, C14408T, A23403G, G25563T, C29353T
**Algeria/EPI_ISL_766873**	GH	B.1	C1059T, C3037T, C10582T, C13335T, C14408T, A23403G, C24937T, G25563T, G25599T, A27965G	**Algeria/EPI_ISL_418241**	GH	B.1	C1059T, C3037T, C10582T, C14408T, C18115T, A23403G, G25563T, C25777T, C26461T, C29353T
**Algeria/EPI_ISL_766867**	GH	B.1.597	C1059T, C3037T, C5144T, T7264C, C7764T, C10279T, C10582T, G10870T, C12367T, C14408T, A23403G, G24236T, G25563T, C27804T, C28830A, C29466T	**Algeria/EPI_ISL_1240721**	G	A	G23012A, A23403G
**Algeria/EPI_ISL_766871**	GR	B.1.1	A949G, C3037T, C14408T, G18677T, T19839C, A23403G, G28881A, G28882A, G28883C, G28903T	**Algeria/EPI_ISL_1240723**	G	A	G23012A, A23403G
**Algeria/EPI_ISL_766864**	GR	B.1.1	C3037T, C14408T, T19839C, A23403G, G28881A, G28882A, G28883C	**Algeria/EPI_ISL_1240725**	G	A	A23063T, C23271A, A23403G
**Algeria/EPI_ISL_766869**	GR	B.1.1	G2305T, C3037T, C14408T, C18928T, A23403G, G28881A, G28882A, G28883C	**Algeria/EPI_ISL_1093430**	G	A	A23063T, C23277T, A23403G
**Algeria/EPI_ISL_766865**	GR	B.1.1	C3037T, C5654T, G12070T, C14267T, C14408T, T19839C, A23403G, G26501C, G28881A, G28882A, G28883C, T29023G	**Algeria/EPI_ISL_1093428**	G	A	C23271A, A23403G
**Algeria/EPI_ISL_766872**	G	B.1	C3037T, C3619T, T6232C, C10582T, C14408T, C17550T, A23403G, T25794C, A26627G, G28774T, C28854T	**Algeria/EPI_ISL_1093427**	Other	A	Del
**Algeria/EPI_ISL_766875**	G	B.1	C3037T, C3619T, C8097T, C14408T, C15480T, A16060C, C17550T, C19017T, A23403G, C28854T	**Algeria/EPI_ISL_1240719**	Other	A	Del
**Algeria/EPI_ISL_766870**	G	B.1	C3037T, C3619T, C8097T, C14408T, C17550T, G19086T, A23403G, C25721T, C28854T	**Algeria/EPI_ISL_1240720**	Other	A	A21717G, C21762T
**Algeria/EPI_ISL_766863**	GH	B.1.36	C3037T, C3619T, C11580T, C14408T, C18877T, C22444T, C22591T, A23403G, G25563T, C26735T, C28854T	**Algeria/EPI_ISL_1240722**	Other	A	A21717G, C21762T
**Algeria/EPI_ISL_766868**	G	B.1	C3037T, C3619T, C5183T, C9430T, C10582T, C14408T, C17550T, G23383A, A23403G, G23868T, C28253T, A28254C, C28744T	**Algeria/EPI_ISL_1240724**	Other	A	Del

**Table 2:** Amino acid replacements detected across different genes in the Algerian sequences.

Query	Gene/Amino Acid Replacement *
**Algeria/EPI_ISL_766874**	NSP2_T85I, NSP3_A1179V, NSP12_P323L, **NSP12_A130V**, NSP12_T85A, Spike_D614G, NS3_Q57H, NS3_S40L, N_A251V
**Algeria/EPI_ISL_766862**	NSP2_T85I, **NSP3_E681D**, NSP12_P323L, NSP12_M629I, Spike_D614G, NS3_Q57H, **NS3_A23T**, N_S186Y
**Algeria/EPI_ISL_766866**	NSP2_T85I, NSP12_P323L, Spike_D614G, NS3_Q57H, N_S186Y
**Algeria/EPI_ISL_766873**	NSP2_T85I, NSP10_A104V, NSP12_P323L, Spike_D614G, NS3_W69C, NS3_Q57H
**Algeria/EPI_ISL_766867**	NSP2_T85I, NSP3_S1682F, NSP12_P323L, Spike_D614G, Spike_A892S, NS3_Q57H, N_S186Y, N_A398V
**Algeria/EPI_ISL_766871**	NSP12_P323L, NSP14_R213L, Spike_D614G, N_M210I, N_G204R, N_R203K
**Algeria/EPI_ISL_766864**	NSP12_P323L, Spike_D614G, N_G204R, N_R203K
**Algeria/EPI_ISL_766869**	NSP2_K500N, NSP12_P323L, NSP14_P297S, Spike_D614G, N_G204R, N_R203K
**Algeria/EPI_ISL_766865**	NSP12_P323L, NSP12_T276M, Spike_D614G, N_G204R, N_R203K
**Algeria/EPI_ISL_766872**	NSP12_P323L, Spike_D614G, N_L167F, N_S194L
**Algeria/EPI_ISL_766875**	NSP3_T1793I, **NSP12_N874H**, NSP12_P323L, Spike_D614G, N_S194L
**Algeria/EPI_ISL_766870**	NSP3_T1793I, NSP12_P323L, NSP14_K349N, Spike_D614G, NS3_A110V, N_S194L
**Algeria/EPI_ISL_766863**	NSP6_T203I, NSP12_P323L, Spike_D614G, NS3_Q57H, N_S194L
**Algeria/EPI_ISL_766868**	NSP3_P822S, NSP12_P323L, Spike_G769V, Spike_D614G, NS8_I121L
**Algeria/EPI_ISL_766861**	NSP12_P323L, Spike_D614G, N_S186Y
**Algeria/EPI_ISL_420037**	NSP2_T85I, NSP3_T1004I, NSP12_P323L, Spike_D614G, NS3_Q57H
**Algeria/EPI_ISL_418242**	NSP2_T85I, NSP12_P323L, Spike_D614G, NS3_Q57H
**Algeria/EPI_ISL_418241**	NSP2_T85I, NSP12_P323L, **NSP14_H26Y**, Spike_D614G, NS3_Q57H, **NS3_L129F**, **E_L73F**
**Algeria/EPI_ISL_1240721**	Spike_D614G, Spike_E484K
**Algeria/EPI_ISL_1240723**	Spike_D614G, Spike_E484K
**Algeria/EPI_ISL_1240725**	Spike_A570D, Spike_D614G, Spike_N501Y
**Algeria/EPI_ISL_1093430**	Spike_T572I, Spike_D614G, Spike_N501Y
**Algeria/EPI_ISL_1093428**	Spike_A570D, Spike_D614G
**Algeria/EPI_ISL_1093427**	Spike_V70del, Spike_H69del
**Algeria/EPI_ISL_1240719**	Spike_Y144del, Spike_V70del, Spike_H69del
**Algeria/EPI_ISL_1240720**	Spike_Y144del, Spike_A67V, Spike_V70del, Spike_H69del, Spike_Q52R
**Algeria/EPI_ISL_1240722**	Spike_Y144del, Spike_A67V, Spike_V70del, Spike_H69del, Spike_Q52R
**Algeria/EPI_ISL_1240724**	Spike_Y144del, Spike_V70del, Spike_H69del

* Replacements in bold indicate unique Algerian mutations.

**Table 3:** Analysis of the unique Amino acid replacements in the Algerian sequences. The percentage of accuracy is indicated between brackets. The Red color stands for deleterious mutation, whereas Green represents neutral mutation.

Mutation	PredictSNP	PhD-SNP	PolyPhen-1	PolyPhen-2	SIFT	SNAP
**NSP12_A130V**	64%	59%	67%	45%	79%	62%
**NSP12_N874H**	72%	58%	74%	60%	79%	62%
**NSP3_E681D**	74%	78%	67%	75%	65%	55%
**NS3_A23T**	55%	72%	59%	54%	53%	50%
**NSP14_H26Y**	75%	83%	74%	87%	87%	71%
**E_L73F**	65%	89%	68%	87%	65%	56%
**NS3_L129F**	72%	68%	59%	68%	45%	72%

## Data Availability

Data used in the current study are available upon request.

## Supplementary Materials

The following are available online at https://www.mdpi.com/article/10.3390/v13081525/s1, Table S1. Details of sequences used in this study. Accession ID, sequence names, date of collection, and country of provenance. Table S2. Estimates of the amino acid replacement on the protein according to Hanada et al. amino acid classification and in reliance on both Grantham and Miyata. G represents Grantham scores, while M represents Miyata scores. Figure S1. Maximum Clade Credibility tree encompassing all available Algeria sequences. The labels are colored, following the continent of origin. Africa is indicated in cyan, Asia in Green, America in Dark blue, Europe in purple, Australia in light brown, and Asia in black. The Algerian sequences are highlighted in Red. Posterior probabilities are indicated at each node as decimal numbers.
