# Supplementary figures and images for: The Algerian Chapter of SARS-CoV-2 Pandemic: An Evolutionary, Genetic, and Epidemiological Prospect
Source: Viruses. 2021 Aug 2;13(8):1525. doi: 10.3390/v13081525 (PMC8402747; doi:10.3390/v13081525)

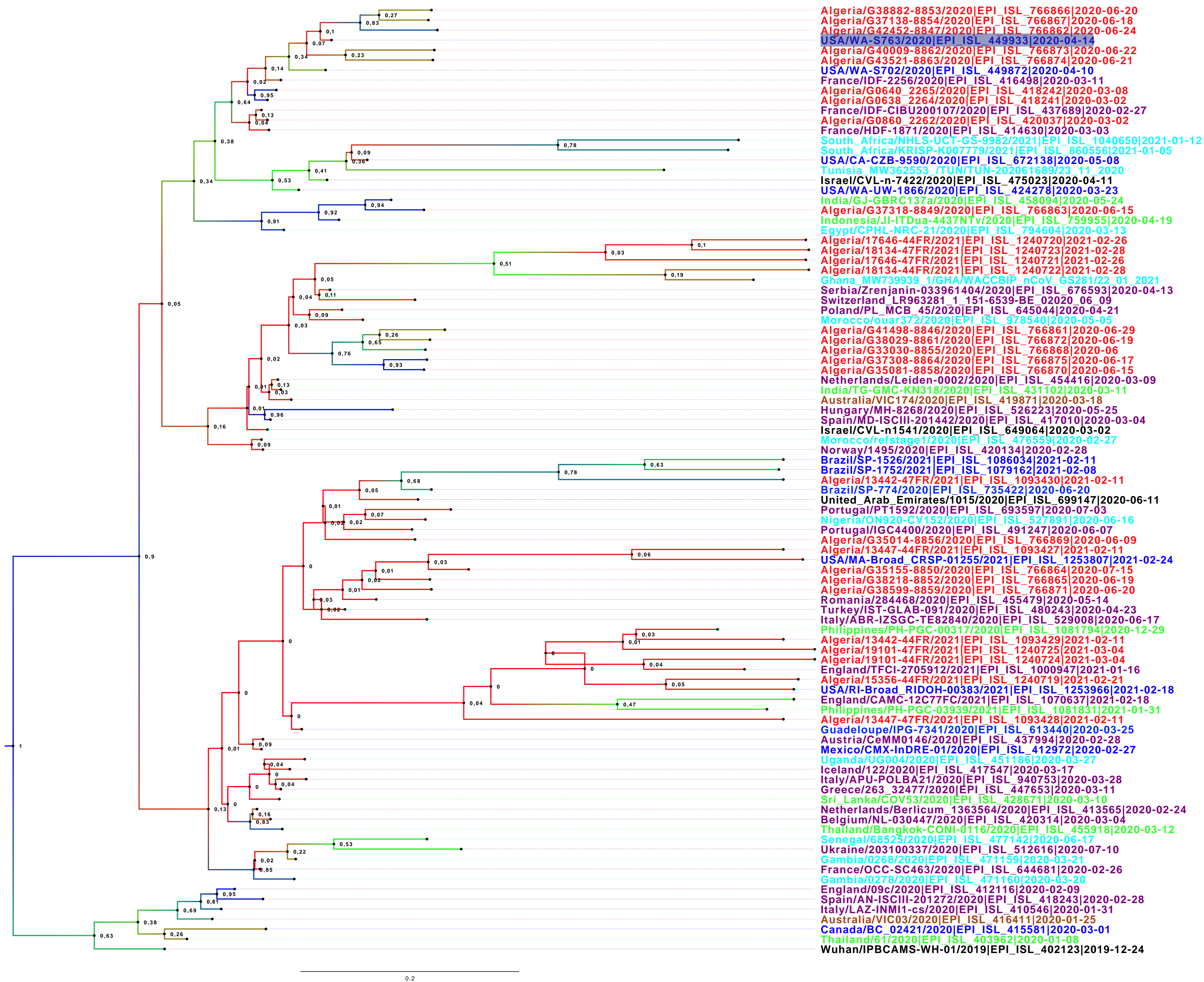

Supplement: Supplementary file 1 [file viruses-13-01525-s001.zip › Fig S1.pdf]
